# Responsive Neurostimulation Targeting the Anterior, Centromedian and Pulvinar Thalamic Nuclei and the Detection of Electrographic Seizures in Pediatric and Young Adult Patients

**DOI:** 10.3389/fnhum.2022.876204

**Published:** 2022-04-12

**Authors:** Cameron P. Beaudreault, Carrie R. Muh, Alexandria Naftchi, Eris Spirollari, Ankita Das, Sima Vazquez, Vishad V. Sukul, Philip J. Overby, Michael E. Tobias, Patricia E. McGoldrick, Steven M. Wolf

**Affiliations:** ^1^New York Medical College, Valhalla, NY, United States; ^2^Department of Neurosurgery, Westchester Medical Center, Valhalla, NY, United States; ^3^Division of Pediatric Neurology, Department of Pediatrics, Maria Fareri Children’s Hospital, Valhalla, NY, United States; ^4^Boston Children’s Hospital Physicians, Hawthorne, NY, United States

**Keywords:** anterior thalamic nucleus, centromedian thalamic nucleus, thalamic stimulation, epilepsy surgery, responsive neurostimulation, intractable epilepsy, RNS, pulvinar

## Abstract

**Background:**

Responsive neurostimulation (RNS System) has been utilized as a treatment for intractable epilepsy. The RNS System delivers stimulation in response to detected abnormal activity, via leads covering the seizure foci, in response to detections of predefined epileptiform activity with the goal of decreasing seizure frequency and severity. While thalamic leads are often implanted in combination with cortical strip leads, implantation and stimulation with bilateral thalamic leads alone is less common, and the ability to detect electrographic seizures using RNS System thalamic leads is uncertain.

**Objective:**

The present study retrospectively evaluated fourteen patients with RNS System depth leads implanted in the thalamus, with or without concomitant implantation of cortical strip leads, to determine the ability to detect electrographic seizures in the thalamus. Detailed patient presentations and lead trajectories were reviewed alongside electroencephalographic (ECoG) analyses.

**Results:**

Anterior nucleus thalamic (ANT) leads, whether bilateral or unilateral and combined with a cortical strip lead, successfully detected and terminated epileptiform activity, as demonstrated by Cases 2 and 3. Similarly, bilateral centromedian thalamic (CMT) leads or a combination of one centromedian thalamic alongside a cortical strip lead also demonstrated the ability to detect electrographic seizures as seen in Cases 6 and 9. Bilateral pulvinar leads likewise produced reliable seizure detection in Patient 14. Detections of electrographic seizures in thalamic nuclei did not appear to be affected by whether the patient was pediatric or adult at the time of RNS System implantation. Sole thalamic leads paralleled the combination of thalamic and cortical strip leads in terms of preventing the propagation of electrographic seizures.

**Conclusion:**

Thalamic nuclei present a promising target for detection and stimulation via the RNS System for seizures with multifocal or generalized onsets. These areas provide a modifiable, reversible therapeutic option for patients who are not candidates for surgical resection or ablation.

## Introduction

Intractable epilepsy, otherwise referred to as drug-resistant epilepsy (DRE), affects up to 30% of all epilepsy patients and often warrants surgical treatment (Engel et al., 2012; Kalilani et al., 2018; Watson et al., 2021). Operative strategies to ameliorate DRE often target thalamic nuclei, including the anterior thalamic nucleus (ANT) and, more recently, the centromedian thalamic nucleus (CMT). The ANT is a common target for localization-related epilepsy due to its connections with the hippocampal outflow tract and Papez circuit (Mirski et al., 1997). The CMT, in contrast, is commonly targeted for multifocal or generalized forms of epilepsy due to its extensive and diffuse connectivity with neocortical regions including the frontal, parietal, frontal, and limbic cortices (Steriade and Glenn, 1982; Lacey et al., 2007). Seizure-like discharges from this location with widespread epileptiform cortical activity and symptomatic seizure activity suggests that the CMT is a potential site for both the propagation and origination of tonic-clonic seizures (Velasco et al., 1989).

While operative resection remains an effective therapeutic option for seizure freedom in DRE, surgical resection is not feasible for a significant cohort of these patients. Circumstances that rule out ablative procedures include scenarios where the zone of ictal onset is not identifiable or in some cases may lie within eloquent areas (Ma and Rao, 2018; Matias et al., 2019; Skarpaas et al., 2019). In these patients, neurostimulation has been approved for treatment in DRE (Nune et al., 2015). Different forms of neurostimulation exist with distinct mechanisms, including vagal nerve, open-loop, and closed-loop systems. Responsive neurostimulation (RNS System, NeuroPace Inc., Mountain View, California) functions through closed-loop stimulation, which continually monitors and stores samples of intracranial EEG at the seizure foci, then delivers therapeutic stimulation upon detection of epileptiform activity (Ma and Rao, 2018; Matias et al., 2019; Skarpaas et al., 2019).

A common strategy for implantation of RNS System leads is to combine cortical strip and thalamic depth leads (“corticothalamic” approach) (Elder et al., 2019; Herlopian et al., 2019; Burdette et al., 2020; Kokkinos et al., 2020; Kwon et al., 2020). While this is done to disrupt epileptogenic network activity at both the sites of origin and spread, there is also uncertainty about the ability to detect electrographic seizures in thalamic nuclei with the RNS System (Burdette et al., 2020). Recent case reports support the detection of electrographic seizures with RNS thalamic leads in the context of Idiopathic Generalized Epilepsy (Kokkinos et al., 2020), Lennox-Gastaut Syndrome (LGS) (Kwon et al., 2020), and Genetic Generalized Epilepsy (Herlopian et al., 2019), among others (Elder et al., 2019; Burdette et al., 2020). Reports of RNS solely targeting thalamic nuclei for focal onset seizures are rare (Kokkinos et al., 2020; Welch et al., 2021). The aim of this study is to supplement the literature by assessing whether detection and treatment of generalized or focal seizures using thalamic RNS leads is broadly applicable to pediatric and young adult epilepsy patients.

## Materials and Methods

The present study is a single center retrospective chart review of patients implanted with thalamic leads in our practice between 2015 and 2022. Fourteen RNS System patients with thalamic lead implantation were identified and their course of treatment were evaluated. Patients with at least one depth lead implanted in a thalamic nucleus, and in whom the lead(s) were subsequently activated, were included. Patients who did not have at least one depth lead both implanted in a thalamic nucleus and connected to the RNS generator for a minimum of 1 week were excluded.

**FIGURE 1:**
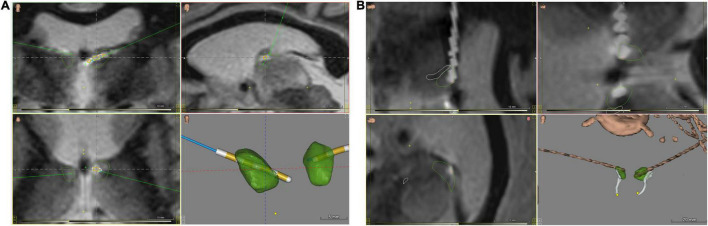
ANT nuclei images with brain atlas overlay of postoperative CT/MRI merge, visualizing implanted RNS depth electrodes. **(A)** Coronal (upper left and lower left) and sagittal (upper right) views, and 3D-rendered image of implanted ANT nuclei without background brain. **(B)** Close-up views of implanted leads from rotated sagittal (upper and lower left) and oblique (upper right) views, along with an oblique view 3D-rendered image of implanted ANT nuclei without background brain. ANT nuclei marked in green, mammillothalamic tracts in white, electrodes in brown. Images generated with WayPoint Navigator version 4.6.6.

**FIGURE 2:**
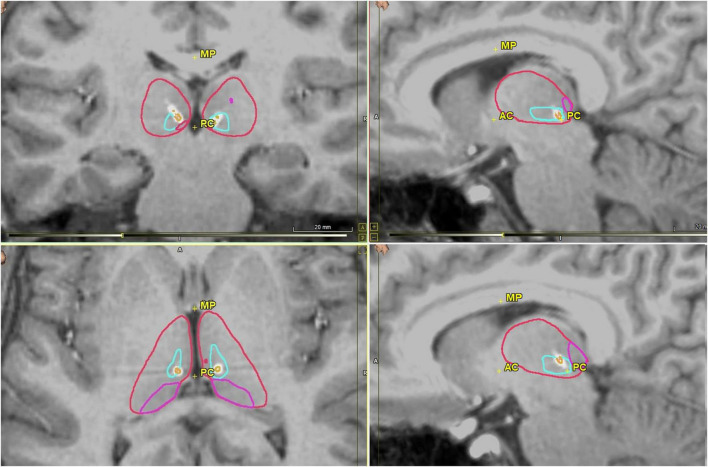
Postoperative CMT nuclei images in coronal (upper left), axial (bottom left) and sagittal views (upper and lower right) with RNS electrodes visualized. CMT is outlined in light blue, thalamus in red, pulvinar in pink, and electrode in white with each contact in orange. Images generated using WayPoint Navigator version 4.6.6.

Contrast-enhanced MRI volumetric sequences were obtained to guide placement of intracranial electrodes using ROSA robotic software (Zimmer Biomet). Patients whose seizure onsets were found to be multifocal or generalized were implanted with RNS depth electrodes in the anterior (Figure 1), centromedian (Figure 2) or pulvinar thalamic nuclei. RNS electrodes were planned using image guided direct targeting on the ROSA robotic planning workstation. FGATIR MRI sequences were used to localize targets in the ANT, while a combination of volumetric T1, contrasted T1 and FLAIR sequences were used to target CM and pulvinar nuclei. Trajectories were planned to optimize parenchymal entry and avoid vasculature using contrast T1 weighted MRI sequences. Strip electrodes were placed with the aid of StealthStation (Medtronic) planning or intraoperative visualization. At the time of surgery, cranial fiducial markers were placed and a stereotactic CT scan was obtained. A CRW headframe was used to attach the patient to the ROSA robotic surgical assistant. The CT and preoperative MR were co-registered to the preoperative electrode trajectory plan. Otherwise, the surgical technique for the robotic placement of the RNS electrodes and generator are substantively similar to those described elsewhere (Tran et al., 2020).

Data on diagnosis and treatment was collected from the electronic medical record system after approval from our Institutional Review Board. The RNS System provides responsive stimulation in response to detections of abnormal intracranial EEG data and stores a continuous record of detections and stimulations, as well as samples of intracranial EEG activity. The activity to be detected and the stimulation parameters are selected by the treating health care provider. Although multiple RNS System leads can be implanted, no more than 2 leads, each of which has 4 electrode contacts, can be connected to the neurostimulator at any one time. Data obtained by the neurostimulator, including intracranial EEG, also referred to as electrocorticography (ECoG) recordings, are obtained by the patient at home, or by the health care provider in the clinic, and transferred over the internet to the NeuroPace Patient Data Management System (PDMS), a secure online repository. Health care providers can review all of their patients’ data on the PDMS. Selected ECoG recordings were manually reviewed in PDMS in patients with a previous or current combination of active cortical and thalamic leads to localize seizure onsets.

As part of routine clinical care, both patients and their caregivers were asked questions about their seizure outcomes by either a NeuroPace engineer, or by the patient’s physician. Patients who had their most recent previous appointment within the past 6 months were asked about the current monthly frequency of debilitating seizures that they experienced, as well as their seizure severity and duration. The frequency of reported debilitating seizures was then compared to patients’ pre-surgical baseline, which had been reported at patient multidisciplinary surgical conferences, and changes were reported in quartiles of 0–24, 25–49, 50–74, and 75–99%. Seizure severity was reported by patients as “Much worse,” “Worse,” “No change,” “Better,” or “Much better.” Changes in seizure duration were reported as shorter episodes, longer episodes, or no change.

### Ethics Statement

Institutional review board approval from New York Medical College was obtained for this study, as was patient consent for the publication of de-identified information.

## Results

### Case Studies

#### Case 1

Patient #1 is a 17-year-old female with a history of infantile spasms beginning at 9 months of age. She was born at 36 weeks gestation by emergency cesarean section for fetal bradycardia and had a subsequent short NICU stay for hypoglycemia and to rule out sepsis. Concurrent electrolyte abnormalities, of calcium and phosphorus, were noted during admission which subsequently resolved by 12 months of age. She has developmental delay and was diagnosed with autism spectrum disorder in 2016. Her current seizure semiologies are as follows: first, focal motor with impaired sensory awareness, wherein she covers her eyes and has eyelid myoclonus, and second, generalized tonic-clonic seizures. Prior to RNS surgery in 2016, she had clusters of seven to eight episodes of the first type of seizure, along with myoclonic jerks of the lower extremities, occurring every 3–4 weeks.

She has tried several antiseizure medications in the past, including phenobarbital, levetiracetam, sodium valproate, felbamate, clobazam, and phenytoin, without relief. She is currently on a regimen of vigabatrin, topiramate, and carbamazepine. In 2011, she underwent a right temporal lobectomy, which included removal of the right parahippocampal gyrus and a right orbito-frontal resection extending back to the right premotor area. Four years later, a complete right frontal disconnection was done, which did not achieve meaningful seizure reduction. Subsequent recordings from subdural grids and strips captured several seizures with onset from the left hippocampus and cingulate, with rapid spread to the bilateral supplemental motor areas and further generalization. RNS was then implanted with depth leads in the left hippocampal gyrus and cingulate cortex, which were activated, as well as bilateral ANT leads which were initially inactive. Postoperative review of her ECoG recordings revealed poor detections from the left anterior cingulate lead, which was then deactivated, and the right ANT lead was activated in its place. Since replacement of her RNS generator in 2020, she has had an average of one seizure per month, for a reduction of 75–99% from her pre-RNS baseline.

#### Case 2

Patient #2 is a 22-year-old male with a history of infantile spasms beginning at 4 months of age. He has severe developmental delay with regression. On physical exam, he is non-verbal and does not ambulate, but grabs with both hands equally. Currently he has two types of seizures: brief seizures with side-to-side eye movements and truncal extension upon awakening, and episodes of tonic seizures with truncal and neck flexion. Prior to RNS surgery, each seizure type occurred on average one to two times per day.

In 2002, spectroscopy showed mildly elevated ventricular lactate, increased choline and decreased NAA in frontal white matter and posterior pons. A PET scan in 2002 showed decreased glucose metabolism in the right hemisphere, localized to the right frontal lobe. In 2004, the scan showed decreased glucose metabolism in the left parietal lobe and bilateral thalami. There was increased perfusion to the left posterior quadrant in the ictal state and normal perfusion to the right frontal lobe interictally. Pathology showed mild cortical disorganization consistent with malformation of cortical development. There was focal subpial gliosis and some of the gyri on the external surface appeared hemorrhagic. Video EEG studies in 2013 demonstrated multifocal epileptiform discharges with shifting predominance of the right and left hemispheres, as well as occasional focal discharges over the right hemisphere preceding events in the left occipital region.

He has tried multiple anti-seizure medications, without relief. He is currently on phenobarbital, lacosamide, felbamate, and gabapentin. From 2006 to 2014 he underwent right frontal lobectomy, corpus callosotomy, VNS placement, anterior commissurectomy, left temporal lobectomy, VP shunt placement, posterior quadrantectomy, and orbito-frontal disconnect. He was briefly seizure-free following corpus callosotomy in 2008, but his symptoms eventually relapsed. As orbito-frontal disconnection failed to achieve meaningful seizure reduction, he had RNS placed in 2015 with left frontal, right temporal and bilateral ANT thalamic leads. Initially, he had right temporal and left frontal leads active, which gave variable response to neurostimulator therapy. In 2017, the left frontal lead was turned off and Left ANT lead was turned on, leaving the right temporal lead active; this produced modest improvement in his seizure frequency for a time, but subsequent worsening led to a second revision in 2020 to bilateral ANT stimulation. At present, his seizures are shorter in duration compared to his pre-RNS baseline, yet largely unchanged in frequency.

### Patient Demographics

The average age at lead implantation was 16.5 ± 5.4 years (Table 1), and nine out of 14 (64.3%) patients had their leads implanted before the age of 18. Primary seizure diagnosis varied, with four patients having LGS. Three patients had known genetic etiologies, which included 22q13 deletion syndrome (also known as Phelan-McDermid syndrome), PRRT2 gene mutation and genetic deletion SCX + DEPDC5. An additional two patients had localization-related epilepsy with secondary generalization, and another two were diagnosed with idiopathic generalized epilepsy.

**TABLE 1:** Patient demographics.

Pt number	Epilepsy diagnosis	Comorbid neurological diagnoses	Seizure semiologies	RNS leads placement	Reasoning for placement	Age at implantation (years)	Prior surgeries
1	Localization related epilepsy with impaired awareness and motor onset	ASD	1. Focal sensory 2. Generalized Tonic Clonic (GTC)	Bilateral ANT + L hippocampus + L cingulate gyrus	Multifocal onset	11	Focal resection
2	LGS—IS	GDD, cerebral palsy	1. Brief seizures with side-to-side eye movements and truncal extension 2. Tonic seizures with truncal and neck flexion	Bilateral ANT + L frontal + R temporal	More robust clinical experience with ANT vs. CMT at time of surgery	14	Focal resection(s), CC and anterior commissurotomy, VPS, VNS
3	Combined generalized and focal:localization related with impaired awareness with secondary generalization and generalized with impaired awareness	Genetic mutation (Phelan-McDermid Syndrome) Arachnoid cyst, ASD	1. Turning blue, stiff, and gurgling then after 1–2 min, starts with body jerking (GTC)	Bilateral ANT + R Anterior Cingulate, R Orbitofrontal	Multifocal onset	14	None
4	Localization-related with secondary generalization, multifocal spikes on previous EEG	LD, dysarthric speech, malformation of cortical development (MCD)	1. Recurrent head drops (atonic seizures)	Bilateral ANT + Bilateral Temporal	Multifocal onset	19	None
5	Localization-related with impaired awareness, focal to bilateral tonic-clonic seizures	Learning Disability	1. Right arm up and flexed- right hand posturing- says “I’m sorry,” “I’m sorry”- confusion 2. Looks distracted, then behavioral arrest—head and eyes to right—movement right arm and leg- grunting sound—hands tremble, head and eyes toward midline	Bilateral CMT + L parietal	Focal onset with failed focal resection	29	Left temporal lobectomy, extension of temporal lobectomy, VNS
6	LGS	ASD	1. Myoclonic Jerks	Bilateral CMT	Generalized seizures	17	VNS
7	LGS, genetic (PRRT2)	ASD	1. Jerks, stares, drops, GTC 2. Brief out of sleep—turn to right, right arm up, left arm crossed	Bilateral CMT	Generalized seizures	16	None
8	LGS—IS	GDD, Mitochondrial Disorder	1. Raises hands, arches legs, raises eyes and smiles 2. Tonic seizures 3. Generalized tonic-clonic 4. Myoclonic jerks	Bilateral ANT + R Mesial Temporal + L Frontal	Widespread onset, not multifocal	10	Corpus callosotomy
9	Generalized Onset	ASD	1. Falls backward onto floor, twists in circles	Bilateral CMT + L Frontal + L Mesial temporal	Generalized seizures	21	None
10	IGE	LD	1. Absence only	Bilateral CMT	Generalized seizures	14	VNS
11	IGE	LD	1. Jerks/head bobs then stare then left hand twitching—able to speak 2. GTC with vomiting- 3. Myoclonic jerks—left hand pain then flaccid	Bilateral ANT + Bilateral parietal	Multifocal or limbic pathways involved	17	None
12	Localization related with impairment of consciousness	Genetic deletion SCX + DEPDC5, LD	1. Arms up and eye dilate—with large myoclonic jerk—last 12 min 2. Bent over and arms out and jerking up lasting for seconds in clusters	Bilateral CMT	Generalized seizures	10	None
13	Juvenile myoclonic epilepsy	LD	1. Absence 2. Myoclonic jerks 3. Focal motor with impaired awareness	Bilateral CMT	Generalized seizures	17	VNS
14	Ohtahara syndrome	Severe developmental delay	1. Head Drops 2. Tonic clonic with fencing posture to either left or right 3. In past—apnea	Bilateral pulvinar	Generalized and multifocal Seizures	13	CC, VNS

*ASD, Autism Spectrum Disorder, GDD, Global Developmental Delay; LD, Learning Disability; IGE, Idiopathic Generalized Epilepsy; CC, Corpus Callosotomy; VPS, ventriculoperitoneal shunt.*

With respect to neurological comorbidities, five patients had Autism Spectrum Disorder and six had a learning disability. Two patients had a comorbid neurological diagnosis of global developmental delay, and one had a concomitant finding of arachnoid cyst. Six (42.8%) patients had no prior surgical history, three (21.4%) had focal resections, three (21.4%) had corpus callosotomy, and six (42.8%) had VNS implant.

### Detections in Anterior Nucleus Thalamic—Bilateral Thalamic and Corticothalamic Leads

**FIGURE 3:**
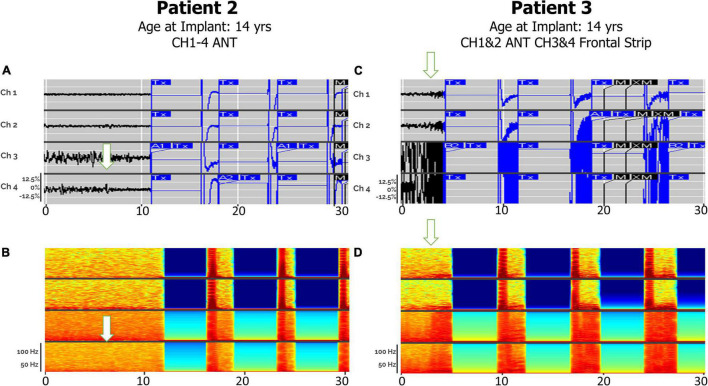
Electrographic seizures detected in ANT (Anterior Thalamic Nucleus), with and without cortical strip leads, recorded by the RNS system. Arrows denote seizure onsets. **(A)** An example of a clinical seizure in Patient 2, stored in the NeuroPace Patient Data Management System (PDMS) over a 30-s window with bilateral ANT leads, left channels (Ch 1. L-ANT1–L-ANT2; Ch 2. L-ANT3–L-ANT4) and right channels (Ch 3. R-ANT1–R-ANT2; Ch 4. R-ANT3–R-ANT4). **(B)** Spectrogram of identical epoch. **(C)** An example of an electroclinical generalized tonic-clonic seizure in Patient 3 stored in PDMS over a 30-s window, with a right ANT depth lead and a right anterior prefrontal cortical strip (Anc) lead. Ch 1. R-ANT1–R-ANT2; Ch 2. R-ANT3–R-ANT4; Ch 3. R-Anc1–R-Anc2; Ch 4. R-Anc3–R-Anc4. **(D)** Spectrogram of identical epoch. Tx, therapy; M, magnet swipe; A1, Pattern A, 1st detector; B2, Pattern B, 2nd detector; M, magnet; XM, magnet removed.

Electrographic seizure onset was detectable via bilateral ANT leads or with a single ANT lead with an additional cortical lead (Figure 3). Patients 2 and 3 both had electrographic seizures, which either originated or propagated through the ANT and cortical regions. Patients 2 and 3 both had electrographic and clinical seizures, which lasted approximately 4 s in duration and either originated or propagated through the ANT and cortical regions. Clinical correlations were obtained at seizure onset by patient-initiated magnet swipes to indicate that a seizure occurred. Patient 2, aged 15 at the time of original RNS System implant, had bilateral ANT leads connected to the neurostimulator, whereas Patient 3, age 14 at the time of RNS System implant, had a right ANT lead and a right anterior prefrontal cortical strip lead connected. Patient 2, aged 15 at the time of original RNS System implant, had bilateral ANT leads connected to the neurostimulator, whereas Patient 3, age 14 at the time of RNS System implant, had a right ANT lead and a right anterior prefrontal cortical strip lead connected. A comparison of the right ANT and right prefrontal cortical strip leads found similar success in detecting the electrographic seizure onset, with a subjectively higher signal-to-noise ratio on spectrographic analysis in the right ANT lead. Spectrogram analysis of the same epochs revealed peak ictal frequencies in ANT at or under 50 Hz.

### Detections in Centromedian Thalamic—Bilateral Thalamic and Corticothalamic Leads

**FIGURE 4:**
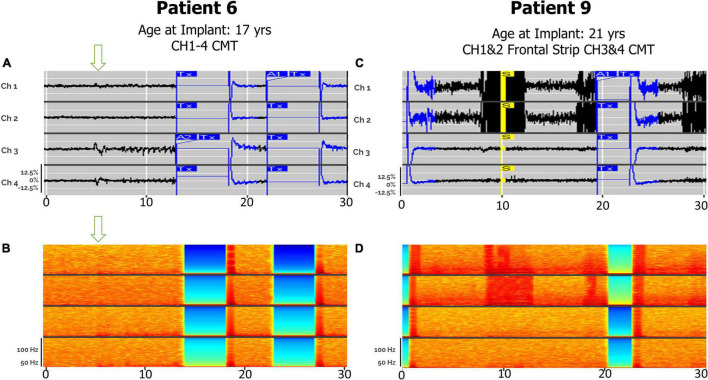
Electroclinical seizures detected in CMT, with and without cortical strip leads, recorded by the RNS system over a 30-s window. Arrows denote seizure onsets. **(A)** An example of an electroclinical seizure consisting of myoclonic jerks in Patient 6, captured with bilateral CMT leads over a 30-s window. Ch 1. R-CMT1–R-CMT2; Ch 2. R-CMT3–R-CMT4; Ch 3. L-CMT1–L-CMT2; Ch 4. L-CMT3–L-CMT4. **(B)** Spectrogram of identical epoch. **(C)** An example of an electroclinical seizure in Patient 9 that was ongoing at the time of capture, detected with left frontal cortical strip (Fnt) + right CMT depth leads over a 30-s window with saturation in the cortex and subsequent spread to CMT thalamic leads. Ch 1. L-Fnt1–L-Fnt2; Ch 2. L-Fnt3–L-Fnt4; Ch 3. R-CMT1–R-CMT2; Ch 4. R-CMT3–R-CMT4. **(D)** Spectrogram of identical epoch. S, Saturation; A2, Pattern A, 2nd detector.

Electrographic seizure detection was also feasible via bilateral CMT leads, or with one CMT and one cortical strip lead (Figure 4), as demonstrated in patients 6 and 9. Each patient had similar 4-s electrographic seizures, which originated or propagated through the CMT nucleus and were correlated with clinical symptoms patient-initiated magnet swipes. Patient 6, who had a primary diagnosis of Lennox-Gastaut Syndrome, demonstrated electrographic seizures that were clinically correlated by patient magnet swipe. Peak ictal frequencies in this patient were less than or equal to 25 Hz in bilateral CMT leads. Seizures were detected with a combination of bandpass and area settings. Patient 9, who had a primary diagnosis of generalized onset epilepsy, had electrographic seizures with peak ictal frequencies in both the frontal cortical and right centromedian depth lead of 100 Hz, approaching the upper limit of detection set by the bandpass filter. Seizures in this patient were detected with bandpass settings alone.

### Detection of Distinct Seizure Types in Thalamic and Hippocampal Lead Sets

**FIGURE 5:**
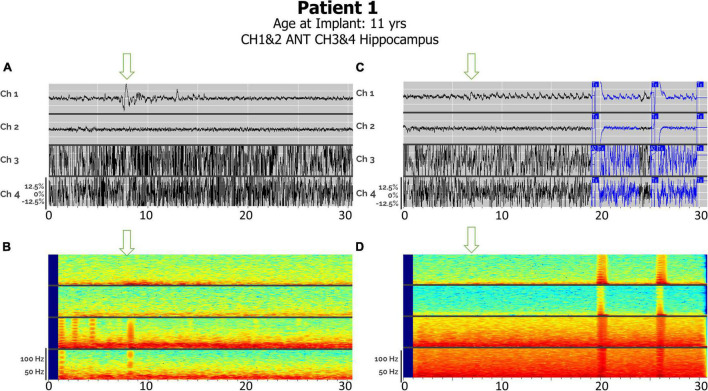
**(A)** Electroclinical detection of a focal sensory seizure over a 30-s window in Patient #1, with arrows denoting seizure onsets in thalamic lead after seizure onset in hippocampus has already started. Ch 1. R-ANT1–R-ANT2; Ch 2. R-ANT3–R-ANT4; Ch 3. L-Hip–1–L-Hip2; Ch 4. L-Hip3–LHip4). **(B)** Spectrogram of same epoch. **(C)** Electroclinical seizure detection and treatment of generalized tonic clonic seizure pattern over a 30-s window with L-Hippocampus onset already started prior to this clip but note thalamic seizure activity onset by the arrow. **(D)** Spectrogram of same epoch. Tx, therapy; T–c, Charge insufficient.

Distinct seizure semiologies were detected in Patient 1 with right ANT and left hippocampal depth leads (Figure 5), with earlier hippocampal involvement in focal sensory seizures with impaired awareness (A), and earlier thalamic involvement in generalized tonic-clonic seizures (C).

### Detection of Seizures in Pulvinar Thalamic Leads

**FIGURE 6:**
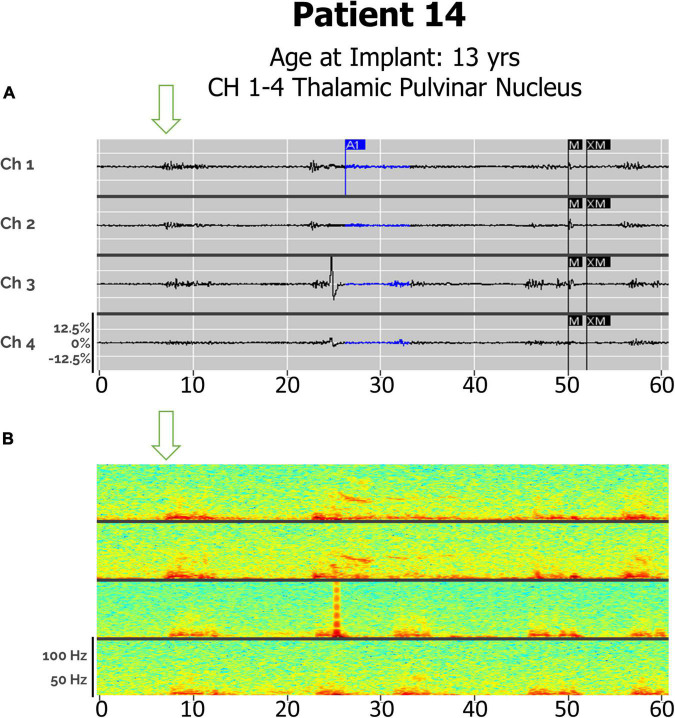
**(A)** Electroclinical seizure detections in Patient 14 captured with bilateral pulvinar (Pulv) leads over an 60-s window, demonstrating both a clinical generalized seizures (Drop), with in-phase waveforms, and focal seizures (left arm fencing posture), with out-of-phase waveforms. Arrows denote seizure onsets. Ch1. L-Pulv1–L-Pulv2; Ch 2. L-Pulv3–LPulv4; Ch 3. R-Pulv1–R-Pulv2; Ch 4. R-Pulv3–R-Pulv4. **(B)** Spectrogram of same epoch.

Distinct seizure semiologies were also detectable in Patient 14 with bilateral pulvinar leads (Figure 6), with generalized seizures detected as in-phase high-frequency discharges, and focal (hemispheric) seizures detected as high-frequency discharges beginning in one lead and spreading to the contralateral lead, in a staggered pattern.

### Detection Settings

All patients with past or current corticothalamic lead sets had seizure onsets originating in cortical regions (Table 2). The most frequently used RNS System detection tool used to detect electrographic seizures in the thalamus was the Bandpass tool. This tool was typically set to detect signal patterns with a minimum frequency of 20 ± 2 Hz sinusoid, a maximum frequency of 125 Hz sinusoid, a minimum amplitude of 0.5–5.0% and a minimum duration of 0.128–1.024 s (*n* = 3). The second most frequently used detection tool was a Bandpass tool with a minimum frequency ranging from 16.79 to 25.57 Hz sinusoid, a maximum frequency of 125 Hz sinusoid, a minimum frequency of 0.78–2.35%, and a minimum duration of 0.128–1.152 s (*n* = 4). Secondary (Pattern B) detection settings were used in 3 out of 14 patients (21.4%) with minimum frequencies of 2–4 Hz Spiking, maximum frequencies of 41.67–62.50 Hz Sinusoid, minimum amplitudes of 5.47–93.84% and minimum duration of 0.512–1.28 s.

**TABLE 2:** Detection settings.

	Lead with earliest detection (if corticothalamic set)	Pattern A													

		1st detector							2nd detector						
		Bandpass				Area			Bandpass				Area		
Pt #	−	Min frequency (Hz)	Max frequency (Hz)	Min amplitude (%)	Min duration (seconds)	Detection threshold (%)	Short-term trend (seconds)	Long-term trend (minutes)	Min frequency (Hz)	Max frequency (Hz)	Min amplitude (%)	Min duration (seconds)	Detection threshold (%)	Short-term trend	Long-term trend
1	−	−−−−−−−−	−−−−−−−−	−−−−−−−	−−−−−−−−	−−−−−−−−	−−−−−−−−	−−−−−−−−	25.57 Sin	125.00 Sin	2.35	0.128	−50.00	2.048	2
2	R Temporal	19.74 Sin	125.00 Sin	0.78	0.512	−−−−−−−−	−−−−−−−−	−−−−−−−−	25.57 Sin	125.00 Sin	0.78	0.384	−−−−−−−−	−−−−−−	−−−−−−
3	R Orbitofrontal	5.02 Sin	125.00 Sin	5.47	1.024	−−−−−−−−	−−−−−−−−	−−−−−−−−	−−−−−−−−	−−−−−−−	−−−−−−−−	−−−−−−−−	−−−−−−−−	−−−−−−−−	−−−−−−−−
4	−	18.15 Sin	125.00 Sin	0.78	0.128	−−−−−−−−	−−−−−−−−	−−−−−−−−	16.79 Sin	125.00 Sin	1.56	0.256	−−−−−−−−	−−−−−−−−	−−−−−−−−
5	−	2.00 Sp	9.62 Sin	2.35	0.768	−−−−−−−−	−−−−−−−−	−−−−−−−−	2.00 Sp	8.33 Sin	3.91	0.768	−−−−−−−−	−−−−−−−−	−−−−−−−−
6	−	−−−−−−−−	−−−−−−−−	−−−−−−−	−−−−−−−−	75.0	2.048	2	1.00 Sp	6.25 Sin	5.47	0.384	——–	——–	−−−−−−−−
7	−	1.00 Sp	11.36 Sin	41.45	0.128	−−−−−−−−	−−−−−−−−	−−−−−−−−	1.00 Sp	15.63 Sin	9.38	0.128	−−−−−−−−	−−−−−−−−	−−−−−−−−
8	L Frontal	1.00 Sp	31.25 Sin	4.69	0.512				19.07 Sin	125.00 Sin	0.78	1.152	−50.00	2.048	2
9	L Frontal	20.83 Sin	125.00 Sin	3.13	0.384	−−−−−−−−	−−−−−−−−	−−−−−−−−	4.61 Sin	20.83 Sin	3.13	0.512	−−−−−−−−	−−−−−−−−	−−−−−−−−
10	−	1.00 Sp	5.43 Sin	9.38	0.128	−−−−−−−−	−−−−−−−−	−−−−−−−−	2.00 Sp	17.86 Sin	8.60	0.256	−−−−−−−−	−−−−−−−−	−−−−−−−−
11	−	−−−−−−−−	−−−−−−−−	−−−−−−−	−−−−−−−−	75.0	2.048	2	−−−−−−−−	−−−−−−−	−−−−−−−−	−−−−−−−−	75.0	2.048	2
12	−	1.00 Sp	15.63 Sin	14.86	0.128	−−−−−−−−	−−−−−−−−	−−−−−−−−	2.00 Sp	12.50 Sin	8.60	0.512	−−−−−−−−	−−−−−−−−	−−−−−−−−
13	−	−−−−−−−−	−−−−−−−−	−−−−−−−−	−−−−−−−−	−−−−−−−−	−−−−−−−−	−−−−−−−−	−−−−−−−−	−−−−−−−−	−−−−−−−−	−−−−−−−−	−−−−−−−−	−−−−−−−−	−−−−−−−−
14	−	1.00 Sp	15.63 Sin	8.60	0.512	−−−−−−−−	−−−−−−−−	−−−−−−−−	41.67 Sin	125.00 Sin	0.78	0.512	−−−−−−−−	−−−−−−−−	−−−−−−−−

**Pattern B**															

	**1st detector**							**2nd detector**							
	**Bandpass**				**Area**			**Bandpass**							
**Pt #**	**Min frequency (Hz)**	**Max frequency (Hz)**	**Min amplitude (%)**	**Min duration (Seconds)**	**Detection threshold (%)**	**Short-term trend (Seconds)**	**Long-term trend (minutes)**	**Min frequency (Hz)**		**Max frequency (Hz)**		**Min amplitude (%)**		**Min duration (seconds)**	

1	38.79 Sin	125.00 Sin	0.78	0.768	12.50	2.048	2	2.00 Sp		41.67 Sin		93.84		0.512	
2	−−−−−−−−	−−−−−−−	−−−−−−−−	−−−−−−−−	−−−−−−−−	−−−−−−−−	−−−−−−−−	−−−−−−−−		−−−−−−−−		−−−−−−−−		−−−−−−−−	
3	−−−−−−−−	−−−−−−−	−−−−−−−−	−−−−−−−−	−−−−−−−−	−−−−−−−−	−−−−−−−−	3.00 Sp		41.67 Sin		35.19		1.28	
4	4.00 Sp	62.50 Sin	6.26	0.384	−−−−−−−−	−−−−−−−−	−−−−−−−−	4.00 Sp		62.50 Sin		5.47		0.512	

*Sin, sinusoid; Sp, spiking.*

### Stimulation Settings

Average burst power was 160 μs, with high variance in burst duration and frequency (Table 3). Therapy limits ranged from 700 to 6,000 bursts per day, with the average daily treatment limit at 3,058 therapies. Four patients (28.6%) were programmed to receive a second burst per therapy. The median charge density was 1.8 μC/cm^2^, with a range of 0.4–4.8 μC/cm^2^. One patient (#11) had all responsive stimulation turned off as he became free of disabling seizures, although detections remain active. Another patient (#13) was only recently implanted, and responsive neurostimulation is currently turned off while detections are being optimized.

**TABLE 3:** Stimulation settings.

		Sequence/Positions		Sequence/Positions		Current		Power		Charge		Duration		Frequency					
		Burst 1		Burst 2		(mA)		(μs)		density (μC/cm^2)		(ms)		(Hz)					
								
Pt number	Number of Stims	Right (1,234)	Left (1,234)	Right (1,234)	Left (1,234)	Burst 1	Burst 2	Burst 1	Burst 2	Burst 1	Burst 2	Burst 1	Burst 2	Burst 1	Burst 2	Daily therapy limit	Ther1 vs. Ther 2	Reset time (minutes)	Witholding
1	5	0000	+ −+−	+−00	0000	3	3	160	80	3	3	100	5,000	200	100	700	Pattern specific	7	Yes
2	5	+ −+−	+−+−	−	−	1	−	160	−	0.5	−	5,000	–	125	–	3,000	Same therapy	7	No
3	5	0000	+ +−−	+−+−	0000	3	1.7	160	160	3	1.7	100	5,000	200	125	3,000	Same therapy	7	No
4	5	+ −+−	+−+−	0000	0000	3	–	160	–	1.5	–	5,000	–	125	–	2,000	Same therapy	7	No
5	5	+ −00	+−00	0000	0000	1.5	0	160	160	1.5	N/A	5000	100	125	200	3,000	Same therapy	7	No
6	5	+ +++	−−−−	−−−−−	+ +++	9.5	9.5	160	160	4.8	4.8	400	400	200	200	6,000	Same therapy	7	No
7	5	+ −+−	+−+−	0000	0000	0.8	–	160	–	0.4	–	5,000	–	125	–	3,000	Same therapy	7	Yes
8	5	+ −00	00+−	0000	0000	1.5	–	160	–	1.5	–	5,000	–	125	–	4,000	Same therapy	7	No
9	5	0000	−−−−	00−−	0000	4	1.3	160	160	2	1.3	100	3,000	200	125	3,000	Same therapy	7	No
10	5	+ −+−	+−+−	0000	0000	1	0	160	160	0.5		5,000	100	125	200	3,000	Same therapy	7	No
11*	5	0000	0000	0000	0000	0	0	160	160	–	–	100	100	200	200	3,000	Same therapy	7	Yes
12	5	+ −00	+−00	0000	0000	0.7	0	160	160	0.7		5,000	100	125	200	3,000	Same therapy	7	No
13*	–	–	–	–	–	–	–	–	–	–	–	–	–	–	–	–	–	–	–
14	5	+ −+−	+−+−	0000	0000	0.5	0	160	–	0.3	–	5,000	–	125	–	3,000	Same therapy	7	No

*Stimulations are turned off.

### Patient Outcomes

The median follow-up time after implantation was 3.1 years (Table 4). Of the 14 patients included in this case series, the most common locations for leads connected to the neurostimulator were bilateral ANT (4/14, 28.6%) and bilateral CMT (6/14, 42.8%). Three patients (21.4%) had one lead in either ANT or CMT and one cortical lead active, and one patient (7.1%) had a combination of right ANT and left hippocampal leads. One patient (7.1%) had bilateral pulvinar leads.

**TABLE 4:** Patient outcomes.

Pt number	Follow-up (Years)	Active RNS leads	Prior active leads	Timing of revision after initial implant (years)	Outcome classification: Seizure frequency	Seizure severity	Seizure duration
1	5	R ANT + L hippocampus	L cingulate gyrus + L hippocampus	3 years, 8 months	75–99%		
2	5.7	Bilateral ANT	L frontal + R temporal, then L ANT + R Temporal	2 years, 4 months and 2 years, 1 month	0–24%	No change	Shorter episodes
3	3.9	R ANT + R anterior cingulate	R orbitofrontal + R ANT	2 years, 9 months	75–99%	No change	Shorter episodes
4	4.3	Bilateral ANT	Bilateral temporal	2 years	0–24%	No change	No change
5	4.5	Bilateral CMT	L parietal	3 years, 11 months	75–99%	Much better	No change
6	0.9	Bilateral CMT	None	–	25–49%	Much better	Shorter episodes
7	0.7	Bilateral CMT	None	–	25–49%	No change	Shorter episodes
8	4.3	Bilateral ANT	L frontal + R mesial temporal	3 years, 8 months	25–49%	Better	No change
9	2.3	R CMT, L frontal	None	–	50–74%	–	Shorter episodes
10	0.2	Bilateral CMT	None	–	25–49%	Better	Shorter episodes
11	4.4	Bilateral ANT	Bilateral parietal	4 years, 10 months	75–99%	No change	–
12	1.4	Bilateral CMT	None	–	0–24%	Worse	Shorter episodes
13	0.02	Bilateral CMT	None	–	–	–	–
14	0.1	Bilateral pulvinar	None	–	–	–	–

No stimulation-related side effects were reported. 12 patients (85.7%) reported improvement in their seizures at last follow-up. Four patients (28.6%) experienced 75–99% improvement in seizure frequency, one patient (7.1%) reported 50–74% improvement in seizure frequency, three patients (21.4%) reported 25–49% improvement, and three (21.4%) reported 0–24% improvement. Two patients (14.3%) had no outcome data available due to the recency of neurostimulator implantation. Four patients (28.6%) reported their seizure severity was “better,” while only one patient (7.1%) reported “worse” seizure severity. In total, seven patients (50%) or their caregivers reported shorter seizure episodes, while three patients (21.4%) reported no change in the duration of their seizures.

Seven patients (50%) underwent revision after the initial implant, on average 3.6 years after the original responsive neurotransmitter had been placed (range = 2.0–4.8 years). Four patients were revised from a combination of ANT and cortical strip (parietal or temporal) to bilateral ANT leads. One patient with cortical dysplasia (Patient 1) underwent revision from left cingulate gyrus to right ANT, with the other active lead (left hippocampus) remaining active.

## Discussion

Our study shows that multifocal and generalized seizures can be reliably detected and treated via RNS thalamic leads alone. To our knowledge, most studies on patients with RNS System thalamic leads utilize a combination of thalamic and cortical leads (Geller et al., 2017; Gummadavelli et al., 2018; Kokoszka et al., 2018; Elder et al., 2019; Herlopian et al., 2019; Burdette et al., 2020; Kwon et al., 2020), with a few exceptions (Kokkinos et al., 2020; Welch et al., 2021). This may be because most clinical expertise in thalamic stimulation has targeted the ANT in the context of deep brain stimulation (DBS); in fact, ANT was chosen as the thalamic lead site for Patient #2 in the present series for this exact reason, as his was the earliest case in our practice. Unlike RNS, which is a closed-loop form of stimulation that allows for the adjustment of stimulation programming in response to patient-specific electrographic seizure patterns (Skarpaas et al., 2019), DBS is open-loop in nature, and cannot record or detect electrographic seizures (Ma and Rao, 2018). As such, uncertainty about the ability to reliably detect seizure activity with thalamic leads may be a partial driver of decisions to place both cortical strip and thalamic depth leads in patients with multifocal or generalized seizures. In addition, we have patients with bilateral thalamic leads, including one patient who clinically recognized a seizure which correlated with a detected electrographic seizure in the thalamus. This corroborates a recent report that thalamic seizure detection can be used for responsive neurostimulation in pediatric patients (Welch et al., 2021). We present the detection settings used in these patients, in hopes of aiding clinical decision-making. While there is currently no FDA approval for RNS treatment in patients under the age of 18, our study corroborates reports of efficacy and safety of RNS in the pediatric population.

Ictal activity in neocortical leads preceded thalamic involvement in all patients with combined neocortical and thalamic lead sets, by visual inspection of their ECoG recordings. A Granger causality analysis of ECoG recordings in Lennox-Gastaut Syndrome (LGS) patients found evidence supporting neocortical precedence (Panov et al., 2020; Welch et al., 2021), and our own findings appear to corroborate this, as two of our patients with corticothalamic lead sets had a primary epilepsy diagnosis of LGS.

In the present study, 11 out of fourteen patients (78.6%) received responsive neurostimulation via bilateral thalamic leads alone, four of which had undergone revision to bilateral ANT from original cortical lead sets. Importantly, RNS System electrographic seizure detection was observed in all 11 of these patients. One of the largest retrospective studies of outcomes in pediatric and young adult patients with drug-resistant epilepsy excluded patients with active bilateral thalamic leads, although patients with a single thalamic lead were included so long as the second lead targeted cortical structures (Nagahama et al., 2021). The focus of that study on FDA-approved indications for RNS therapy, which requires patients to have ≤ 2 seizure foci, likely accounts for their exclusion of patients with bilateral thalamic leads. An even larger retrospective review of pediatric RNS patients included a single patient with bilateral ANT leads connected and active, although six other patients had a combination of thalamic and cortical leads implanted and active.

Leads revisions, when they occurred, were done to improve therapeutic responses in patients who did not respond robustly to initial responsive neurostimulation treatment. The need for leads revision surgery is not considered an adverse event, as it is an anticipated outcome; patients are implanted with up to four leads during the initial RNS surgery, although only two can be connected to the generator at one time, to facilitate future possible leads revision procedures. However, since all surgical procedures carry a degree of risk, patients in our practice were followed and evaluated for a minimum of 2 years before any leads revisions were considered (Table 4). Newer versions of the RNS generator will have the capacity to connect up to four leads at once, obviating the need for revision in most cases.

While the ANT and CMT are frequently used targets for responsive neurostimulation, the pulvinar is a less common target (Burdette et al., 2021). Patient 14, the first patient with Ohtohara syndrome to be implanted with an RNS device, was implanted with bilateral pulvinar leads, as his seizures were alternating between multifocal and generalized, and presurgical planning using ROSA robotic software indicated that this was the most accessible thalamic target. A recent case series examining responsive neurostimulation in patients with idiopathic generalized epilepsy described one patient with absence seizures with eyelid myoclonus, in whom a pulvinar contact was used for initial seizure detection but not stimulation (Sisterson et al., 2022). We have been able to detect both types of his seizure semiologies, with phase-locked detections across all leads representing generalized seizures and hemispheric onsets corresponding to multifocal events (Figure 6).

While there is currently no FDA approval for RNS treatment in patients under the age of 18, our study corroborates reports of efficacy and safety of RNS in the pediatric population (Panov et al., 2020; Welch et al., 2021). None of the patients in the present study experienced any adverse events necessitating either discontinuation of RNS therapy or corrective surgery. Moreover, no stimulation-related adverse effects were noted in our patient population. 36% of the patients in this study reported greater than 50% reduction in their seizure burden, the majority (4/5) of whom were under the age of 18 at the time of RNS system implantation. Multiple factors appear to have influenced this responder rate. The fact that more than one third of patients had a follow-up period under 1 year is likely a major contributor, as the increased efficacy of responsive neurostimulation therapy over time is well-documented (Bergey et al., 2015; Nair et al., 2020). Encouragingly, our two patients with bilateral CMT leads with greater than 2 years’ follow-up experienced > 50% decreases in seizure frequency, echoing recent reports in patients with idiopathic generalized epilepsy with bilateral CMT leads (Sisterson et al., 2022). It should also be noted that the frequency and severity of patients’ seizures typically change in cyclic patterns (Karoly et al., 2021), wherein environmental and other external factors, as well as internal physiologic fluctuations, modulate a patient’s seizure risk and response to antiseizure therapies. We corroborate these cyclic changes in response in several of our own patients. However, since the scope of the present study focused more on seizure detections than outcomes, we chose to report the most conservative and up-to-date outcomes for each patient. The data gathered support the idea that thalamic neurostimulation can improve quality-of-life in patients with multifocal and generalized onset forms of drug-resistant epilepsy.

There are limitations of the present study that must be acknowledged, including a lack of objective comparison of the relative detecting abilities of bilateral thalamic vs. combined cortical and thalamic lead sets, such as a signal-to-noise ratio calculation. This was not possible under the constraints of the Patient Data Management System, although it may be feasible using raw electrocorticography data. Comparisons of this kind would likely need to compare the same patients before and after lead revision, as the clinical characteristics and RNS detection settings unique to each patient complicates any attempt at comparing groups of patients. Another limitation is the relatively diverse patient population with regards to primary epilepsy diagnosis. Although this allows us to claim the ability to detect seizures in thalamic leads across the spectrum of epileptic disorders, the numbers of patients with each diagnosis are too small for meaningful statistical comparison.

Given the demonstrated ability to detect and treat electrographic seizures at thalamic targets, we expect that bilateral thalamic RNS will eventually become a standard of care for intracranial neurostimulation for many forms of generalized or multifocal drug-resistant epilepsy.

## Conclusion

Here, we have demonstrated the detection of electrographic seizures in the anterior, and centromedian and pulvinar thalamic nuclei, in both pediatric and adult patients with drug-resistant epilepsy. Bilateral thalamic implantation offers comparable seizure detection as does cortical and thalamic or sole cortical lead sets. This ability to detect seizures from thalamic nuclei permits a new approach to the detection and treatment of multifocal and generalized epilepsy.

## Data Availability Statement

The original contributions presented in the study are included in the article/Supplementary Material, further inquiries can be directed to the corresponding author/s.

## Ethics Statement

The studies involving human participants were reviewed and approved by the New York Medical College IRB. Written informed consent to participate in this study was provided by the participants’ legal guardian/next of kin.

## Author Contributions

CM, PM, and SW contributed to conception and design of the study. MT, PO, CM, VS, PM, and SW generated the data used in the present study. CB and AN performed data extraction and analysis, and prepared figures and tables. CB wrote the first draft of the manuscript. CB, AN, ES, SV, VS, CM, and AD wrote sections of the manuscript. CB and AD prepared the manuscript and associated files for submission. All authors contributed to manuscript revision, read, and approved the submitted version.

## Conflict of Interest

SW and PM reports honoraria from LivaNova, Eisai, UCB, Sunovion, Greenwich Pharmaceuticals, and participation as an investigator in clinical trials for Zogenix, GW Pharma, NeuroPace, Neurelis, UCB, Eisai. CM reports speaker fees from LivaNova, and participation as an investigator in clinical trial for NeuroPace. The remaining authors declare that the research was conducted in the absence of any commercial or financial relationships that could be construed as a potential conflict of interest. The handling editor GW declared a past co-authorship with the author(s) SW and PM.

## Publisher’s Note

All claims expressed in this article are solely those of the authors and do not necessarily represent those of their affiliated organizations, or those of the publisher, the editors and the reviewers. Any product that may be evaluated in this article, or claim that may be made by its manufacturer, is not guaranteed or endorsed by the publisher.
