# Supplementary material for: Responsive Neurostimulation Targeting the Anterior, Centromedian and Pulvinar Thalamic Nuclei and the Detection of Electrographic Seizures in Pediatric and Young Adult Patients
Source: Front Hum Neurosci. 2022 Apr 12;16:876204. doi: 10.3389/fnhum.2022.876204 (PMC9039390; doi:10.3389/fnhum.2022.876204)
Supplement: Supplementary file 1 [file Presentation_1.PPTX]

## Slide 1
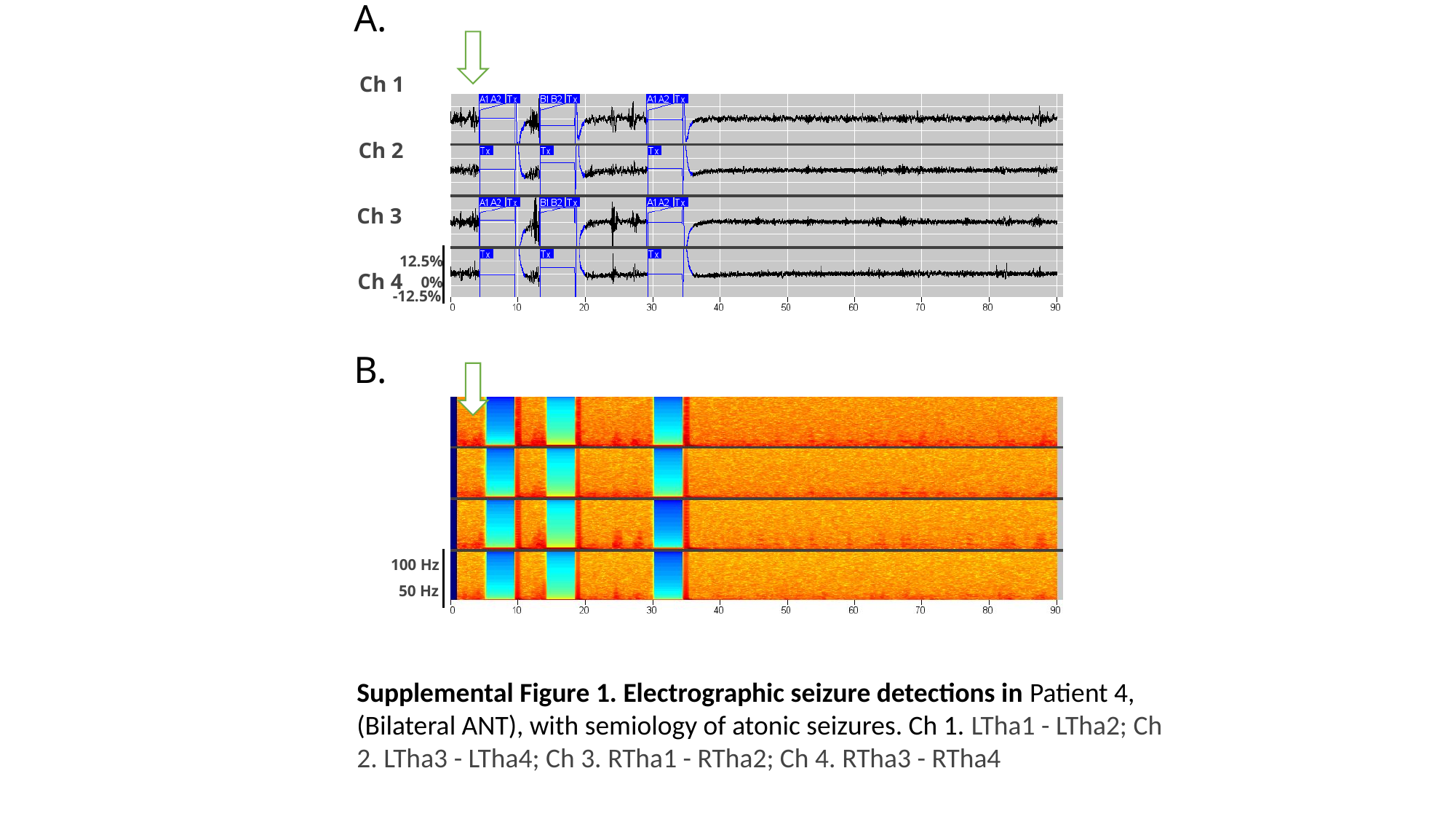

A.
Ch 1
Ch 2
Ch 3
12.5%
Ch 4
0%
-12.5%
B.
100 Hz
50 Hz
Supplemental Figure 1. Electrographic seizure detections in Patient 4, (Bilateral ANT), with semiology of atonic seizures. Ch 1. LTha1 - LTha2; Ch 2. LTha3 - LTha4; Ch 3. RTha1 - RTha2; Ch 4. RTha3 - RTha4

## Slide 2
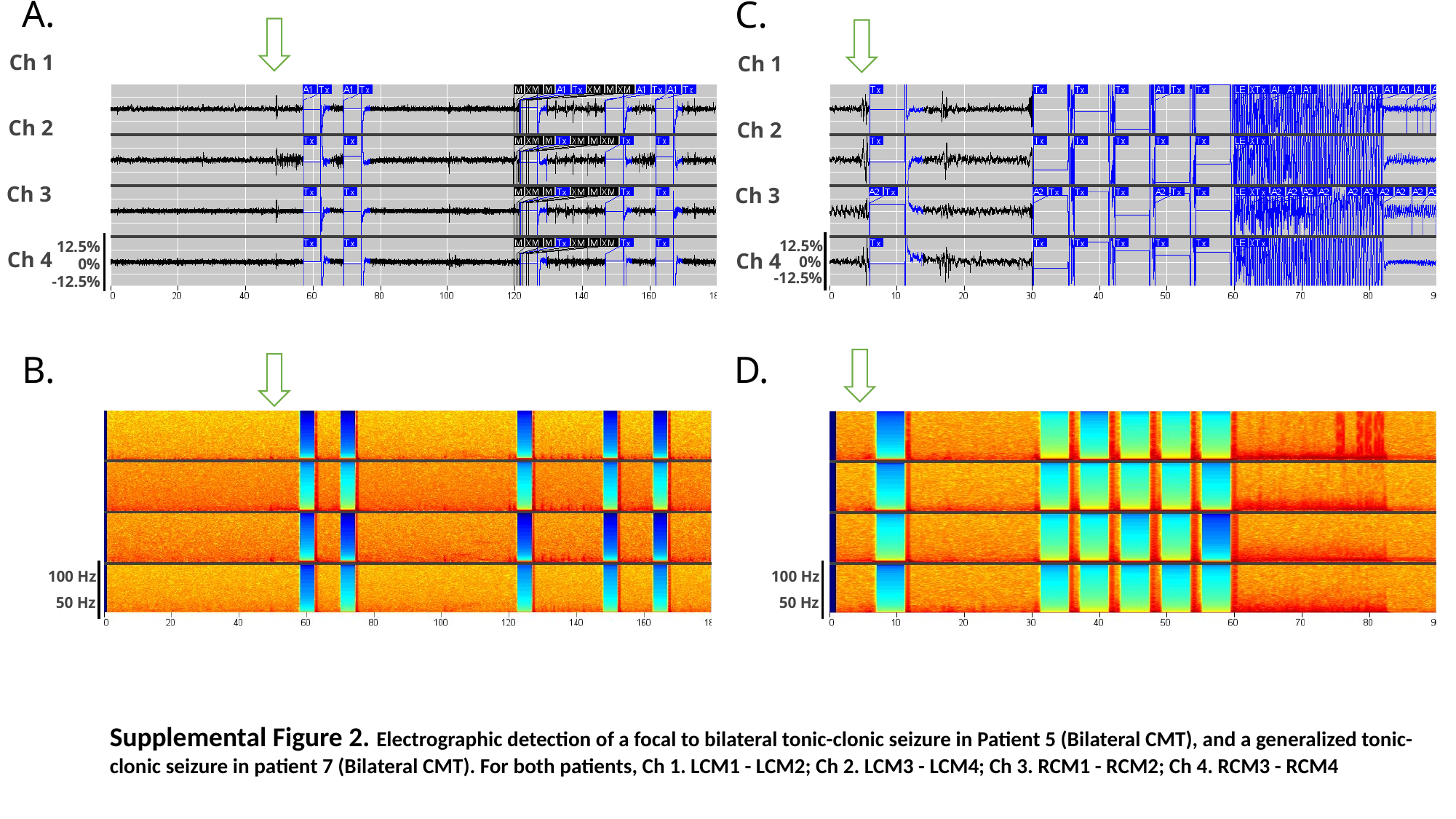

A.
C.
Ch 1
Ch 1
Ch 2
Ch 2
Ch 3
Ch 3
12.5%
12.5%
Ch 4
Ch 4
0%
0%
-12.5%
-12.5%
D.
B.
100 Hz
100 Hz
50 Hz
50 Hz
Supplemental Figure 2. Electrographic detection of a focal to bilateral tonic-clonic seizure in Patient 5 (Bilateral CMT), and a generalized tonic-clonic seizure in patient 7 (Bilateral CMT). For both patients, Ch 1. LCM1 - LCM2; Ch 2. LCM3 - LCM4; Ch 3. RCM1 - RCM2; Ch 4. RCM3 - RCM4

## Slide 3
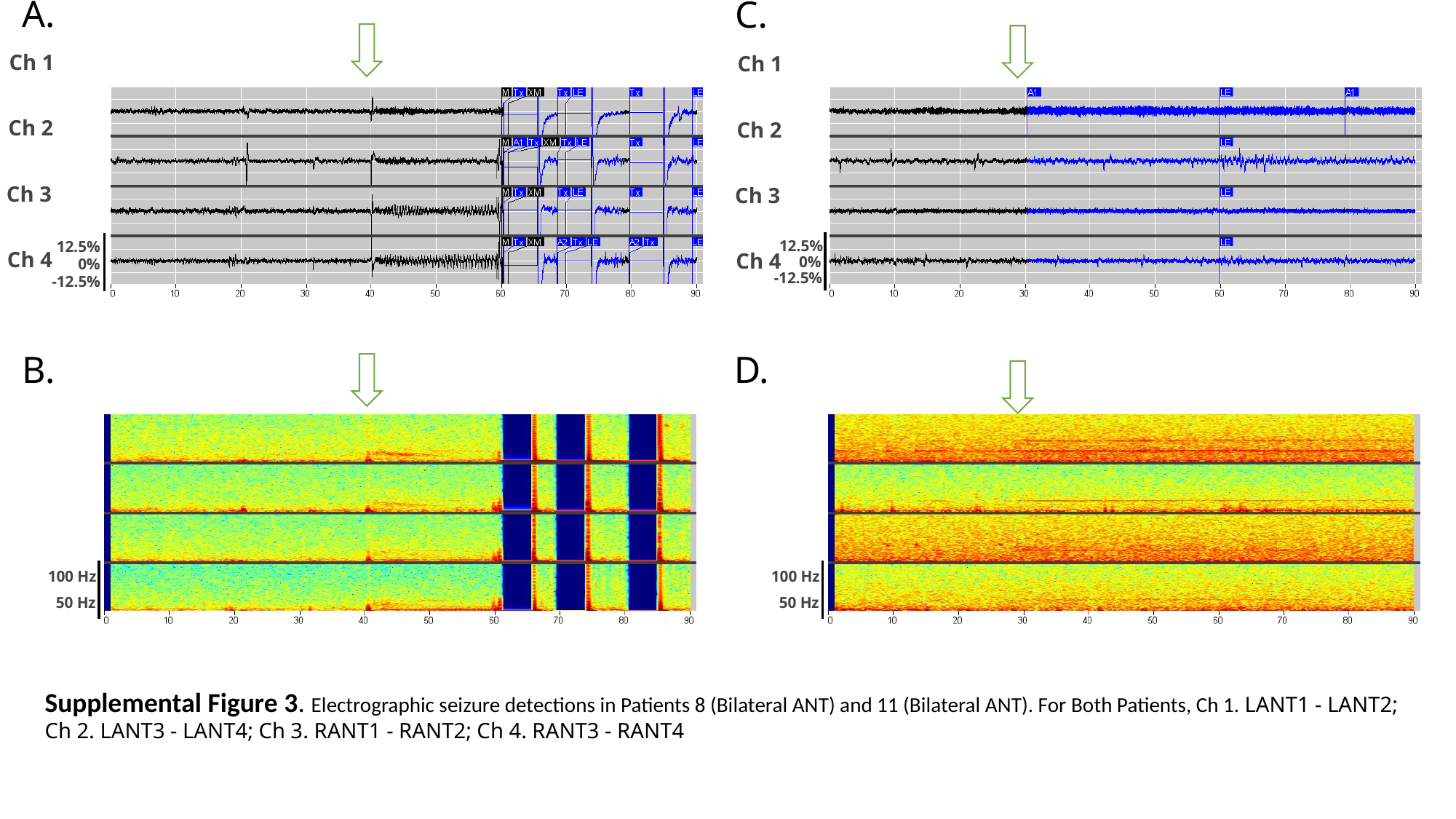

A.
C.
Ch 1
Ch 1
Ch 2
Ch 2
Ch 3
Ch 3
12.5%
12.5%
Ch 4
Ch 4
0%
0%
-12.5%
-12.5%
D.
B.
100 Hz
100 Hz
50 Hz
50 Hz
Supplemental Figure 3. Electrographic seizure detections in Patients 8 (Bilateral ANT) and 11 (Bilateral ANT). For Both Patients, Ch 1. LANT1 - LANT2; Ch 2. LANT3 - LANT4; Ch 3. RANT1 - RANT2; Ch 4. RANT3 - RANT4

## Slide 4
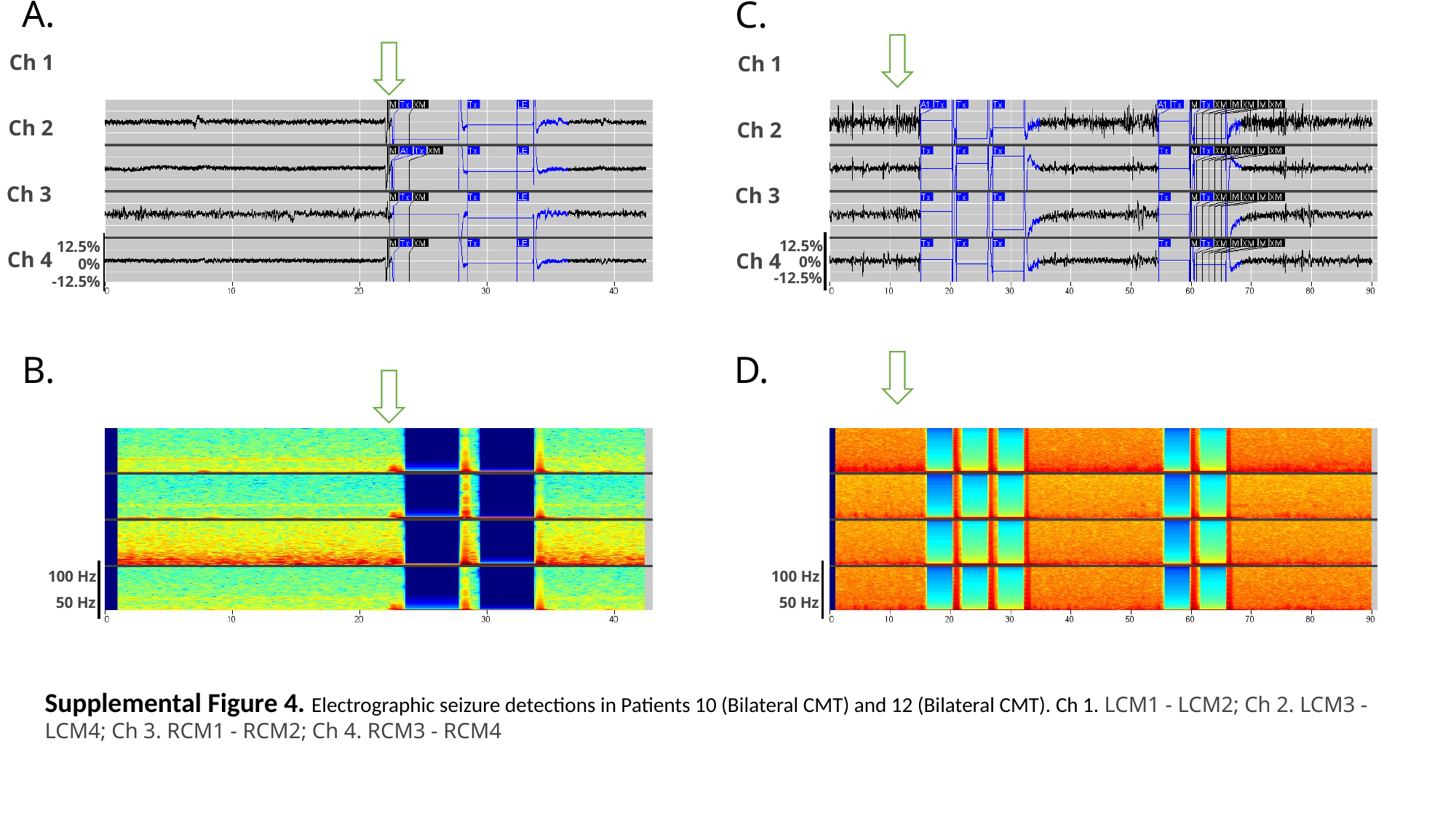

A.
C.
Ch 1
Ch 1
Ch 2
Ch 2
Ch 3
Ch 3
12.5%
12.5%
Ch 4
Ch 4
0%
0%
-12.5%
-12.5%
D.
B.
100 Hz
100 Hz
50 Hz
50 Hz
Supplemental Figure 4. Electrographic seizure detections in Patients 10 (Bilateral CMT) and 12 (Bilateral CMT). Ch 1. LCM1 - LCM2; Ch 2. LCM3 - LCM4; Ch 3. RCM1 - RCM2; Ch 4. RCM3 - RCM4

## Slide 5
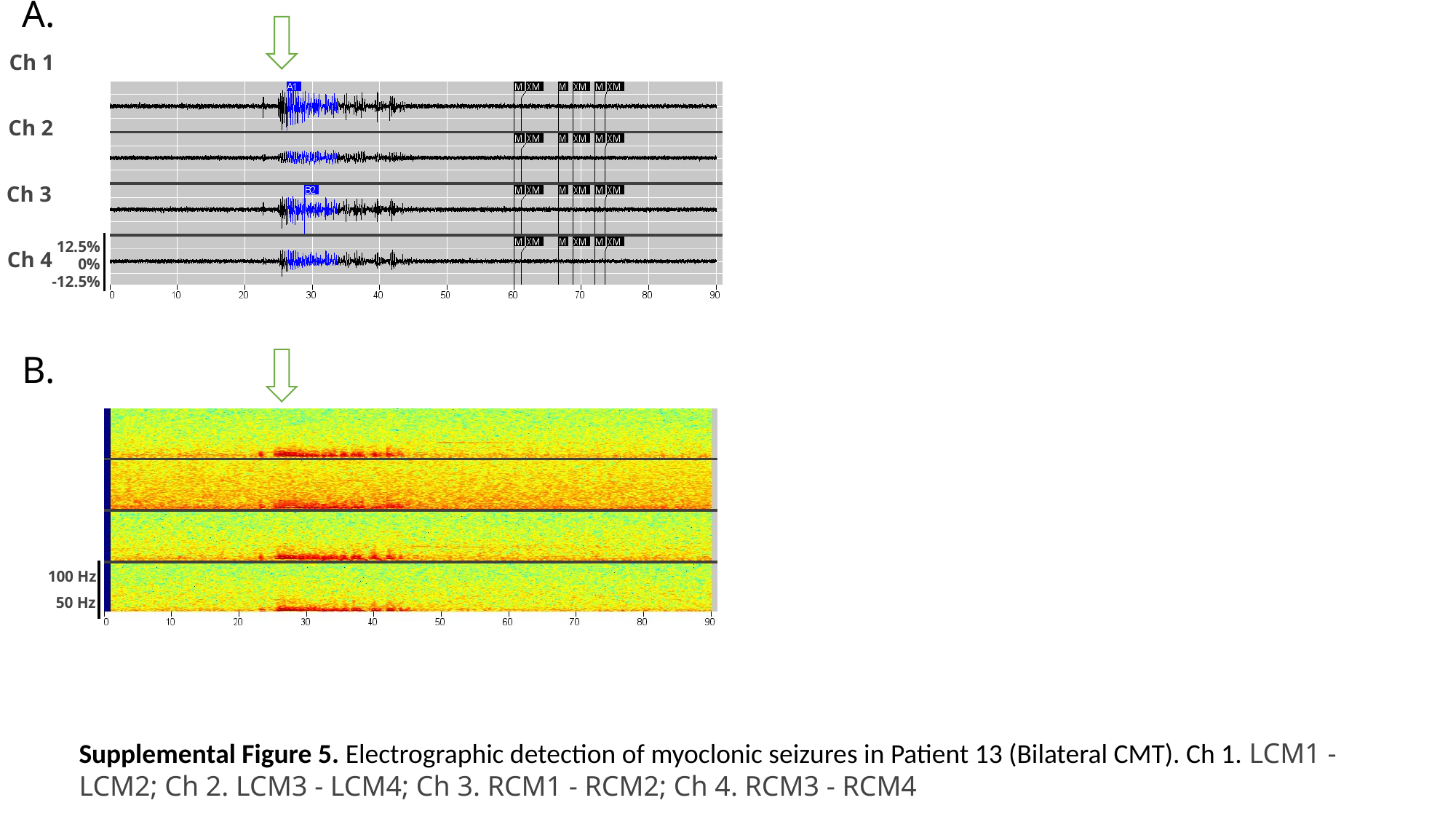

A.
Ch 1
Ch 2
Ch 3
12.5%
Ch 4
0%
-12.5%
B.
100 Hz
50 Hz
Supplemental Figure 5. Electrographic detection of myoclonic seizures in Patient 13 (Bilateral CMT). Ch 1. LCM1 - LCM2; Ch 2. LCM3 - LCM4; Ch 3. RCM1 - RCM2; Ch 4. RCM3 - RCM4
